# Cardiac cycle efficiency as prognostic index in ICUs

**DOI:** 10.1186/cc9475

**Published:** 2011-03-11

**Authors:** A Donati, S Loggi, C Scorcella, MR Lombrano, L Botticelli, MC Melia, A Carsetti, R Domizi, S Tondi, P Pelaia

**Affiliations:** 1 Università Politecnica delle Marche, Ancona, Italy

## Introduction

Cardiac cycle efficiency (CCE) can be calculated by the pressure recording analytical method (PRAM), a mini-invasive pulse-contour system that can provide beat-to-beat monitoring of cardiac output [1]. CCE is a new parameter that ranges from -1 to +1, with -1 being the worse and +1 the best possible performance of the cardiac cycle in terms of hemodynamic balance maintenance [2]. These characteristics make CCE a possible prognostic index, especially in critical patients who often present hemodynamic instability.

## Methods

We recruited 157 consecutive patients admitted to the ICU undergoing hemodynamic monitoring, and the following parameters were registered in the first 24 hours from the admission: hemodynamic parameters (cardiac index, d*p*/d*t*_max _and CCE) detected from the MostCare monitor (based on the PRAM algorithm), PaO_2_/FiO_2 _ratio, arterial lactates, SAPS II. We also divided the patients into seven diagnostic categories and take note of the outcome.

## Results

We inserted all data into the logistic regression analysis model. The significant variables that take place in the regression equation included: SAPS II (*P *< 0.0001), lactates (*P *= 0.033), d*p*/d*t*_max _(*P *= 0.032) and the diagnostic category (*P *= 0.020). CCE was not significant and was not included in the model. See Table 1.

**Table 1:** Results of logistic regression analysis

Variable	Significance	Odds ratio
d*p*/d*t*_max_	0.032	0.191
SAPS II	0.0001	1.174
Diagnostic category	0.020	
Lactates	0.033	1.018

## Conclusions

We demonstrate that CCE registered in the first 24 hours from admission is not a good prognostic index. The differences of CCE value between patients with good and negative outcome was not statistically significant. This result may suggest that a low CCE value in 24 hours from admission does not necessarily mean a bad outcome but, on the contrary, can be successfully improved by a therapeutic approach. It will be interesting to study whether there are some correspondences between CCE variations and modifications of the clinical conditions of the patients that may predict a positive or negative outcome.
